# Scoring docking conformations using predicted protein interfaces

**DOI:** 10.1186/1471-2105-15-171

**Published:** 2014-06-06

**Authors:** Reyhaneh Esmaielbeiki, Jean-Christophe Nebel

**Affiliations:** 1 Department of Statistics, University of Oxford, 1 South Parks Road, Oxford OX1 3TG, UK; 2 Faculty of Science, Engineering and Computing, Kingston University London, Kingston-Upon-Thames, Surrey KT1 2EE, UK

**Keywords:** Protein-protein interaction, Interface prediction, Homology modelling, Docking, Model scoring, Model ranking

## Abstract

**Background:**

Since proteins function by interacting with other molecules, analysis of protein-protein interactions is essential for comprehending biological processes. Whereas understanding of atomic interactions within a complex is especially useful for drug design, limitations of experimental techniques have restricted their practical use. Despite progress in docking predictions, there is still room for improvement. In this study, we contribute to this topic by proposing T-PioDock, a framework for detection of a native-like docked complex 3D structure. T-PioDock supports the identification of near-native conformations from 3D models that docking software produced by scoring those models using binding interfaces predicted by the interface predictor, Template based Protein Interface Prediction (T-PIP).

**Results:**

First, exhaustive evaluation of interface predictors demonstrates that T-PIP, whose predictions are customised to target complexity, is a state-of-the-art method. Second, comparative study between T-PioDock and other state-of-the-art scoring methods establishes T-PioDock as the best performing approach. Moreover, there is good correlation between T-PioDock performance and quality of docking models, which suggests that progress in docking will lead to even better results at recognising near-native conformations.

**Conclusion:**

Accurate identification of near-native conformations remains a challenging task. Although availability of 3D complexes will benefit from template-based methods such as T-PioDock, we have identified specific limitations which need to be addressed. First, docking software are still not able to produce native like models for every target. Second, current interface predictors do not explicitly consider pairwise residue interactions between proteins and their interacting partners which leaves ambiguity when assessing quality of complex conformations.

## Background

Since proteins function by interacting with other molecules, analysis of protein-protein interactions is essential for comprehending biological processes. Given that alternation in those interactions can result in diseases, their identification is key information for drug design. For example, discovery that the Von Hippel-Lindau syndrome (VHL), a disorder characterised by the formation of tumours and cysts, is caused by a single mutation in the VHL protein which perturbes binding to the hypoxia-inducible factor has led to the manufacture of novel cancer drugs [1-3]. Experimental techniques such as Y2H [4], phage display [5] and affinity purification [6] have played an important role in deciphering protein interaction networks. Despite these efforts only 10% of the human interactome has been experimentally determined [7]. Moreover, elucidation of biological processes often requires an understanding of atomic interactions within a complex. Although such information may be generated by X-ray crystallography or nuclear magnetic resonance, high costs in time and resources, and technical limitations have prevented their wide spread usage. Since approximately 40,000 protein complexes are available in the Protein Data Bank (PDB) [8] and PQS [9], they can be used for computational modelling of interactions [10]: docking intends to predict a complex 3D structure from the structures of its components. Using energy-based cost functions, it explores the space of possible conformations and generates a list of plausible models. Although it often contains near-native conformations, additional knowledge, such as binding site location or interacting residues, is required to identify them. As a consequence, accurate prediction of protein interfaces has become an important component of a docking framework [11-14]. After a review of interface predictors, we explore how they have been used as constraint to evaluate docking conformations.

### Protein-protein interface prediction

Computational methods which have been proposed for identifying interface residues of proteins can be broadly divided into two non-exclusive categories based on their use of protein information. The first approach is based on the specific features of sequences and/or structures, while the second one explores proteins which are either sequentially or structurally related to the query protein (QP).

A large variety of intrinsic features have been used for interface prediction, they include composition and propensity of interface residues [15], physico-chemical properties [16,17], predicted structural characteristics [16], secondary structure [18], solvent-accessible surface area [19,20], geometrical shape of the protein surface [19] and crystallographic B-factor [18,21]. One of the first studies was conducted by Ofran and Rost [15] which used amino-acid composition to predict interfaces. Since they had previously shown that residues at interface have a totally different composition than others [22], this information was used to train a Neural Network (NN). They further improved their approach by introducing ISIS [16,23] which uses both evolutionary profiles and predicted structural features for NN training. Better performance, especially in terms of sensitivity, demonstrates the value of integrating predicted structural information. ISIS prediction of a few residues (low sensitivity) with high accuracy suggests the importance of these residues in binding which have been referred as hot-spots residues. Other studies have confirmed the intuitive assumption that inclusion of structural information improves performance since non-surface residues can be trivially eliminated [19,24]. A popular approach has been to exploit that information, either predicted or actual, using machine learning methods. Whereas Cons-PPISP relied on consensus predictions from multiple neural networks [25], ProMate, adopted an approach using a Bayesian network involving 13 different features [26]. Eventually, the usage of additional structural information in the form of side chain energy estimation allowed PINUP performing better than both Cons-PPISP and ProMate [27]. Finally, a meta predictor, meta-PPISP, which combines the scores of PINUP, Cons-PPISP and Promate, was shown to outperform each of these individual methods [24,28].

An alternative research line has exploited the fact that structurally similar proteins (or structural neighbours) share similar interaction sites even if they are unrelated [10,29,30]. PredUs extracts structural neighbours of the QP, maps interface residues onto the QP and scores these residues using a SVM based classifier according to their intrinsic features [29,30]. PrISE follows a similar approach but using only local structural similarity from a repository of structural elements [31]. Experiments show PredUs and PrISE perform similarly and outperform meta-PPISP [31,32]. Despite homology requirements potentially reducing the scope of usability of prediction methods, many approaches have exploited available homologous structures and/or sequences [33,34]. These methods use Multiple Sequence Alignment and/or phylogenetic tree to detect homologues and extract evolutionary information. HomPPI divides the homologues of a QP into three zones according to interface conservation: Safe, Twilight and Dark Zones. Interfaces are then predicted using an MSA of homologues from the most reliable available zone [34]. Performance was significantly improved by using Structure-based-MSA (S-MSA) in IBIS [35]. IBIS combines sequence and structure conservation scores to detect potential binding sites. IBIS structurally aligns QP with its homologues creating an S-MSA which highlights the interface residues of homologues. Then, using the S-MSA a binding site similarity matrix is generated by comparing the structure and sequence of each homologue against all other homologues. Using the matrix, similar binding sites are clustered into groups which are ranked according to a weighted combination of sequence similarity score and conservation score. The inferred binding site of the best rank group is then mapped onto the QP. Recently, we introduced a novel template based approach, WePIP, which goes further than any method in exploiting homology [32]: not only continuous scoring is used to express homology closeness to the QP, but the nature of interaction partners is also considered. Initial evaluation has suggested that WePIP outperforms competitors in terms of precision and accuracy [32].

### Scoring protein-protein docking conformations

Protein-protein docking aims to computationally predict the 3D structure of a protein complex using the unbound structures of its components (useful reviews can be found in [36-39]). Docking algorithms can be divided into two groups [40], i.e. template-based [10,41,42] and template-free docking. With the increase in the number of 3D structures template-based docking has become particularly popular using experimentally determined structures as templates to generate new complexes. Template-based docking is particularly attractive since, unlike template-free docking, its low computational cost makes it suitable for interactome scale predictions. Template-free docking still remains highly important since not all proteins can be modelled using templates, [43]. In addition, free-docking approaches with their refinement stage have made it possible to generate high resolution structures [43] which are important for understanding the molecular mechanism of protein contacts. In this paper we simply refer to template-free docking as ‘docking’.

Performances of docking algorithms are compared biannually in the CAPRI (Critical Assessment of Predicted Interaction) competition [44] and are evaluated against larger protein docking benchmarks [45-47]. Those algorithms explore thousands of docking orientations (sampling) that are assessed using an energy-based scoring function [38] involving, in the case of ZDOCK [48], measures of shape complementary, desolvation and electrostatics. In order to introduce flexibility, ensemble of conformations [39] have also been used to generate docking conformations. These ensembles are taken from X-ray or NMR structures or generated using computational methods such as (Molecular Dynamic) MD simulations, normal modes and loop modelling. One way of docking ensembles is to dock them one by one (cross-docking) but since it is computationally expensive methods such as mean-field approach have been used [49]. Two studies of Smith et al. [50] and Grünberg et al. [51] investigated the use of ensembles docking by using MD simulations along with 3D-DOCK and HEX docking methods. Although they discovered an increase in the number of native like solutions in the pool of docked conformations, scoring became more difficult since wrong solutions were given higher ranks. In order to introduce flexibility and to reduce the size of the sampling space, some methods have adopted energy minimization (EM) techniques such as MD [13,52] or Monte Carlo [53-56] simulated annealing.

These methods still produce a large number of solutions which require post-processing to detect native-like conformations. One should also be aware that since the present techniques neglect the presence of water during docking, the assembly of models can differ from the actual targets within a soluble environment [57]. In order to refine the list of putative docking models, an additional step may be performed by applying energy minimisation, clustering or knowledge generated from available 3D structures. Typically, Cluspro [58-60], a state-of-the-art method, clusters the top 1000 models in terms of energy to generate a shorter set (hundreds) of model representatives. Although these models are associated with scores, they have shown to be unreliable to identify near native configurations [61].

Since docking software produce 100’s to 1000’s of putative models, their exploitation requires the ability to score them accurately [62-64]. Intuitively, physical-based scoring functions are particularly attractive since they can be applied to any model by exploiting physiochemical features of the atoms. ZRANK [63] relies on the usage of a combination of three atom-based terms, i.e. van der Waal, electrostatics and desolvation. In order to handle conformational changes upon binding, an extension of ZRANK, IRAD, integrated residue and atom based potentials [64]. Experiments showed it outperforms ZRANK when dealing with complexes of medium docking difficulty.

Since comparative studies have shown that energy-based scoring functions are error-prone [65,66], machine learning and knowledge–based statistical methods seem to be more promising approaches. Zhoe et al. [67] proposed a supervised (SVM) and a semi-supervised (TSVM) feature-based learning method trained using 3D interface features generated from interaction interfaces of protein complexes. Experiments revealed that both approaches can distinguish between native and non-native structures with accuracy around 80%. More recently, Othersen et al. [68] conducted a similar experiment using mutual information to select discriminative structural features [46]. They identified 11 of them which led to good identification of near-native models.

Knowledge of interface residues has proved particularly successful [69] and has been applied to either constrain the initial search space of docking software [13,14] or score docking conformations by calculating the similarity between the interfaces of the docked models and the predicted ones [69,70]. Since evaluating interfaces can be applied to models generated by any docking software and can be combined with other scoring function, it has proved more popular and practical. Experiments aiming at gaining insight into the value of using interface knowledge showed that knowledge of at least 40% of interface residues is sufficient to significantly improve ZDOCK rankings [24]. As a consequence standard interface prediction approaches, such as cons-PPISP [25], Promate [26] and HomPPI [34], were extended to evaluate the fit of docked proteins against their predicted binding sites [69,70]. By combining five interface predictors, i.e. Promate [26], PPI–Pred [71], PPISP [72], PINUP [27], and a predictor based on NN [73] into one framework called metaPPI [12], success rates were improved by 15% in comparison to the best individual predictors. Finally, instead of representing interacting interfaces as a two-patch system, SPIDER [74] evaluates multi-residue interactions using a library of contacts containing graph representations of common interfacial patterns. Although SPIDER has claimed to outperform ZRANK, its usage is limited by the requirement of accurate and high resolution interfaces.

### Overview

As highlighted in the latest edition of CAPRI [75], despite progress in docking predictions, there is still room for improvement. In this study, we contribute to this topic by proposing T-PioDock (Template based Protein Interface prediction and protein interface Overlap for Docking model scoring), a framework for detection of a native-like docked complex 3D structure. T-PioDock aims at supporting the identification of near-native conformations from 3D models produced by docking software by scoring those models. As supported by the review in the “Scoring protein-protein docking conformations” section, T-PioDock exploits template based predictions of complexes’ binding interfaces to evaluate docking configurations.

**Figure 1:**
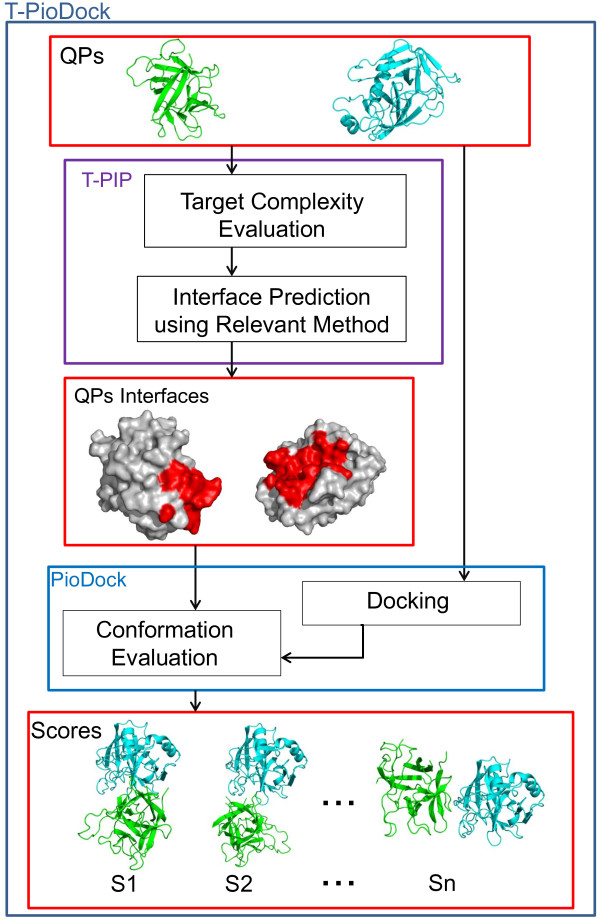
**T-PioDock pipeline.** T-PIP evaluates target complexity and predicts their interfaces using the most relevant method; PioDock exploits these interfaces to score models produced by standard docking.

T-PioDock’s pipeline is described in Figure 1. The input to the system is the 3D structures of the query proteins. First, the T-PIP module (Template based Protein Interface Prediction) evaluates the complexity of the protein targets –i.e. ‘trivial’ , ‘homologous’ or ‘unknown’- in terms of homologue availability in the PDB [8] and predicts their interfaces using the most appropriate template-based method (see ‘T-PIP: Template based Protein Interface Prediction’ in Methods section). These interfaces are then passed to the PioDock module (Protein Interface Overlap for Docking model scoring) which exploits them to score conformation models produced by docking software. Finally, those scores can be used to help at the identification of near-native conformations by ranking available conformation models.

In this paper, we first conduct an exhaustive evaluation of interface predictors on a set of standard benchmark datasets and demonstrate that the T-PIP methodology whose predictions are customised to target complexity performs best. Second, we provide a comparative study between T-PioDock and other state-of-the-art scoring methods on the most complete docking benchmark dataset. This establishes T-PioDock as the best performing approach. Then, we discuss those results in the context of identification of the best conformations. Finally, we present the methodology behind T-PIP and T-PioDock.

## Results

### Datasets and tools

Interface predictors and docking model ranking approaches are evaluated using three standard benchmark datasets: Ds56unbound [44], Docking Benchmark 3.0 (DBMK3.0) [47] and Docking Benchmark 4.0 (DBMK4.0) [76]. These datasets contain high-resolution protein structures both in their unbound and bound forms.

Ds56unbound is comprised of 56 unbound chains generated from 27 CAPRI targets, T01 ~ T27 [44]. In total, it contains 12173 residues including 2112 interacting ones. This dataset is used to perform evaluation of all interface prediction methods of interest.

**Figure 2:**
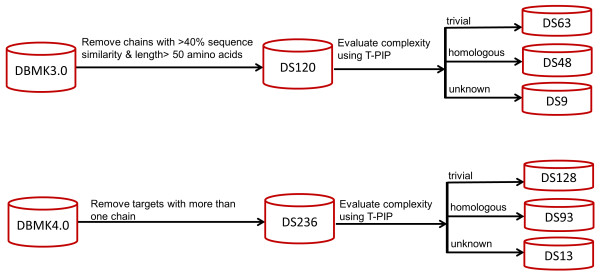
**Creation of DS120 and DS236.** Processing of DBMK3.0 and DBMK4.0 to create relevant evaluation datasets.

DBMK3.0 and DBMK4.0 were originally introduced for the evaluation of protein docking methods. DBMK3.0 contains 124 unbound-unbound targets and 309 protein chains, whereas DBMK4.0 is an extension with 53 new targets. Targets are classified into three main groups, i.e. enzyme/inhibitor, antibody/antigen and other categories, and three categories, i.e. rigid body, medium difficulty and difficult - based on their degree of conformational change between the bound and unbound forms. These datasets contain contains two-body (hetero-dimeric) targets where the individual elements can consist of dimers, trimmers and tetramers rather than monomers. Since there is no agreed methodology for the evaluation of predicted interfaces when dealing with complexes involving more than two chains, those oligomers were excluded from our experiments to ensure fair and consistent comparisons. Moreover, in order to allow comparison with PredUs, we only considered a subset of DBMK3.0, where the chains share at most 40% sequence similarity and their lengths are above 50 amino acids. As a consequence, we produced two subsets of DBMK3.0 and DBMK4.0, i.e. DS120 and DS236 (See Additional files 1 and 2 for details), with 120 and 236 chains respectively. The most promising interface prediction methods were further evaluated on those datasets and docking experiments were conducted on DS236. In this paper, these datasets are further divided into ‘trivial’ , ‘homologous’ or ‘unknown’ categories (see ‘T-PIP: Template based Protein Interface Prediction’ in Methods section) to allow comparisons between methods (see Figure 2). For example, DS93 is a subset of DS236 which contains 93 protein chains which belong to the ‘homologous’ category. Similarly, DS128 comprises 128 chains from the ‘trivial category’.

In this study, initial docking predictions were produced using the ClusPro 2.0 docking server [60], which performed best at CAPRI 2009 [58]. For a pair of proteins, Cluspro generates hundreds of conformational models usually containing at least one near native model. These models are generated by minimising their energy and are then clustered. Clusters are ranked based on their size. Unfortunately, these rankings have proved unable to detect the near-native models [61,70].

### Evaluation of interface prediction methods

In the first set of experiments, performance of state-of-the-art methods was performed using the Ds56unbound dataset. According to T-PIP, 27 chains were classified as ‘trivial’, 24 as ‘homologous’ and 5 as ‘unknown’ based on homologues availability in the PDB (see ‘T-PIP: Template based Protein Interface Prediction’ in Methods section). In addition, to evaluate interface prediction without knowledge of the QP structure, we also produced results where the QP sequence, instead of its structure, was aligned with a Structure-based-MSA (S-MSA) of its homologues. Those results are labelled as T-PIP_QPseq+S-MSA_. Since the IBIS server may provide several interfaces for a given protein, performance is calculated here by selecting the interface with the highest score. Note that two targets could not have their interface predicted using IBIS (5HMG-A and 5HMG-B). It should be stressed that, although T-PIP_QPseq+S-MSA_ does not requires the actual QP structure, it relies on the availability of the 3D structure of QP homologues.

Based on results on Ds56unbound in Table 1 template based approaches, i.e. IBIS, PrISE, PredUs and T-PIP, perform better than feature based methods. Comparison between these two classes of approaches is also available on DS120 in Table 2. In general, template-based methods show a better recall score, while feature-based methods display a better precision score. This suggests that feature-based methods predict a smaller set of the ground truth interfaces, but are more accurate in that prediction. This is especially important for mutagenesis studies.

**Table 1:** Evaluation of interface prediction methods using the Ds56unbound dataset

**Predictor (*DS56unbound)***	**Precision**	**Recall**	**F1**	**Accuracy**	**MCC**
Promate	28.7	27.3	28.0	76.6	14.0
PINUP	30.4	30.1	30.2	76.9	16.4
Cons-PPISP	37.4	34.5	35.9	79.5	23.8
Meta-PPISP	38.9	24.0	29.7	81.1	20.2
IBIS	48.2	29.3	34.4	82.5	27.9
PrISE	43.7	44.0	43.8	81.2	32.6
PredUs	43.3	**53.6**	47.9	73.2	30.4
**T-PIP**	**53.8**	48.5	**49.6**	**84.0**	**41.1**
**T-PIP**_**QPseq+S-MSA**_	53.4	48.1	49.2	83.9	40.7

Best results are shown in bold.

**Table 2:** Comparison of interface predictors’ performance on DS120 and DS236

**Predictor & dataset**	**Precision**	**Recall**	**F1**	**Accuracy**	**MCC**
**T-PIP DS120**	**52.6**	56.1	**52.5**	**85.4**	**45.1**
PredUs DS120	47.3	**58.2**	48.5	69.4	24.4
PrISE DS120	38.5	48.9	40.9	80.7	31.2
PINUP DS120	40.7	34.7	37.5	78.3	24.6
IBIS DS120	42.6	37.4	37.4	83.8	29.9
Cons-PPISP DS120	46.5	30.6	36.9	80.4	26.7
Meta-PPISP DS120	49.0	26.7	34.6	81.1	26.2
Promate DS120	36.5	30.3	33.1	77.1	19.5
**T-PIP DS236**	**53.2**	**55.3**	**52.1**	**85.3**	**44.8**
PrISE DS236	41.2	47.5	41.5	81.0	32.0
IBIS DS236	40.9	36.9	36.2	83.6	28.8

Best results are shown in bold.

Moreover in Table 1, T-PIP displays either best or second best results competing with PrISE [31] and PredUs [29,30] depending on the metric considered. Comparison between standard T-PIP and T-PIP_QPseq+S-MSA_ suggests that availability of the QP structure only marginally increases performance and is, therefore, not required for interface prediction. Nevertheless standard T-PIP is used in all remaining experiments.

Further tests were conducted on the best performing approaches, i.e. IBIS, PrISE, PredUs and T-PIP, using the DS120 and DS236 datasets. Note that for DS120 PredUs and IBIS failed to process 1 (1ZK0-B) and 9 proteins, respectively. For DS236, while IBIS failed to make predictions for 32 proteins which did not have ‘close’ homologues, i.e. at least 30% sequence similarity to the QP and 75% binding site overlap with the QP structure, T-PIP, which investigates remote homologues, was only unable to process 2 proteins (1H20-A and 1QFD-A) that do not have any structural neighbour. Since PredUs used DS120 chains for training, its performance on an independent dataset is likely to be lower (results on DS236 were not available). When using the PrISE server, query chains were removed from the database used for computation of similar structures.

As shown in Table 2, T-PIP also displays the best performance on DS120 and DS236. Interestingly, T-PIP displays similar performance on Ds56unbound, DS120 and DS236 even though DS236 contains more structures from the difficult and medium-difficulty categories. Table S1 and Table S2 of Additional file 3 provide comparisons between T-PIP, PredUs and PrISE on enzyme-inhibitor, antibody-antigen and others categories of the DBMK datasets. Based on those tables, T-PIP performs better in enzyme-inhibitor and others categories. Although no definite conclusion can be drawn for the antibody-antigen category, since there are very few chains, the T-PIP method is unlikely to perform well since it relies on creating MSA of homologues, whereas formation of anti-body–antigen complexes makes difficult the creation of meaningful MSAs [77]. Table 3 displays T-PIP results in each of those categories. As expected, better performance is achieved when targets have fewer conformation changes upon binding.

**Table 3:** T-PIP performance on DS120 and DS236 according to DBMK categories

**Predictor & Categories**		**Precision**	**Recall**	**F1**	**Accuracy**	**MCC**
**T-PIP DS120**		52.6	56.1	52.5	85.4	45.1
	**Rigid Body (86chains)**	57.1	61.3	57.3	86.7	50.7
	**Medium-Difficulty (18 chains)**	42.0	50.8	44.5	84.5	35.9
	**Difficult (16 chains)**	42.9	34.0	35.8	79.2	26.2
**T-PIP DS236**		53.2	55.3	52.1	85.3	44.8
	**Rigid Body (156 chains)**	56.8	59.4	56.2	86.7	49.5
	**Medium-Difficulty (44 chains)**	45.1	52.2	47.0	85.6	39.3
	**Difficult (34 chains)**	46.9	37.6	38.5	78.4	28.6

Experiments also confirm that homology information benefits interface prediction. As seen in Table 4, interfaces for the ‘homologous’ category display higher quality than those for the ‘unknown’ category: although recall performance remains stable (the method used for processing the ‘unknown’ category, PredUs, has a particularly good recall, see Table 2), F1 and accuracy measures are better by around 10%, precision by 15% and MCC by a third. Prediction performance of the ‘trivial’ category is also provided in Table 4 for the sake of being exhaustive. However, that category is not the target of this paper. Since the ‘homologous’ category is our main target, and we have selected WePIP instead of PredUs to make prediction for this category, we have compared WePIP performance with PredUs on ‘homologous’ category of DS120 (As shown in Table 3 and Figure 1, this dataset is named DS48). Results showed in Table 5 show a mixed picture where WePIP performs better on DS48 in terms of accuracy and MCC, while PredUs displays higher precision, recall and F1. Moreover, in 50% of cases, WePIP performance based on F1 score is equivalent or better than PredUs. As consequence, we cannot conclude about the superiority of a method above the other for the ‘homologous’ category. However, one should be aware that, since PredUs was trained with a dataset including DS48 targets, its performance may be overestimated. Moreover, while PredUs require the 3D structure of the QP WePIP can predict interfaces using only the QP sequence which makes WePIP more applicable. WePIP is clearly an important state-of-the-art approach for protein interface prediction.

**Table 4:** T-PIP performance on DS120 and DS236 according to target complexity

**Predictor & Categories**		**Precision**	**Recall**	**F1**	**Accuracy**	**MCC**
**T-PIP DS120**		**52.6**	**56.1**	**52.5**	**85.4**	**45.1**
	**Trivial DS63**	64.9	67.5	66.0	89.1	60.5
	**Homologous DS48**	36.5	43.7	38.2	82.9	29.6
	**Unknown DS9**	31.6	41.8	34.5	73.3	19.2
**T-PIP DS236**		**53.2**	**55.3**	**52.1**	**85.3**	**44.8**
	**Trivial DS128**	65.2	63.8	62.3	88.6	57.0
	**Homologous DS93**	39.7	44.9	40.3	82.5	31.1
	**Unknown DS13**	32.3	46.3	36.6	74.1	22.1

In DSx, x in the number of chains in the category.

T-PIP results are shown in bold.

**Table 5:** WePIP performance compared to PredUs on DS48

**Predictor & Categories**	**Precision**	**Recall**	**F1**	**Accuracy**	**MCC**
**T-PIP DS48**	37.2	43.8	38.3	82.7	29.9
**PredUs DS48**	38.1	55.5	42.1	68.8	20.6

**Figure 3:**
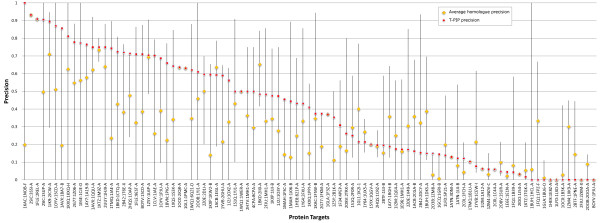
**Interface precision of ‘homologous’ targets in respect to available homologues.** Horizontal line connects the maximum and minimum precisions calculated for homologues of a given target. Average homologue precision and T-PIP precision are shown by yellow diamonds and red squares, respectively.

**Figure 4:**
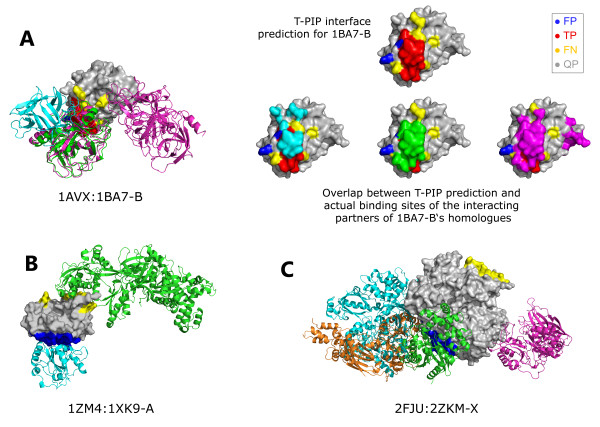
**Examples of successful and failed interfaces predicted by T-PIP. A)** 1AVX:1BA7-B, **B)** 1ZM4:1XK9-A and **C)** 2FJU:2ZKM-X. Query chains are displayed in grey using solid surface representation. Representatives of interacting partners of proteins homologous to QPs are displayed as cartoons. Red, yellow and dark blue patches on solid surfaces represent correctly (TP), missed (FN) and wrongly (FP) predicted surface residues, respectively. In A) cyan, green and pink patches correspond to the actual binding sites of the interacting partners of 1BA7-B‘s homologues.

Processing of ‘homologous’ targets by WePIP relies on extracting the relevant interacting residues from the interfaces of homologous proteins. In order to evaluate this process, for each protein from the 93 ‘homologous’ targets defined in Table 2 (DS93- See Additional file 4), the precision that would have been obtained using simply the interface of a homologue is computed. This shows how much the interface of a given homologue complex is representative of the solution binding site. In addition, for a given target, the average of its homologues precisions and its T-PIP precision is calculated. Figure 3 shows the quality of T-PIP predictions with respect to target homologues. Note that query proteins are identified using their association to their target employing the following notation: ABCD:WXYZ-E, where ABCD is the PDB code of the complex target and WXYZ-E is the query protein PDB code-chain, e.g. 1ZM4:1XK9-A.In most cases the quality of T-PIP predictions is above average which confirms its ability to extract relevant information from a homologue set. However, the figure also reveals that T-PIP is unable to improve on the best homologue interface. We analyse some of the targets in more detail. First, we detail a successful case, 1AVX:1BA7-B, where T-PIP extracts binding information from a set of 14 homologous complexes – only 3 representatives are illustrated in Figure 4A. Using appropriate ligand and QP weighting (see ‘T-PIP: Template based Protein Interface Prediction’ in Methods section), T-PIP manages to predict quite accurately the interface of the target. Second we focus on a couple of cases where T-PIP performed extremely badly. Figures 4B and 4.C explain prediction failures for 1ZM4:1XK9-A and 2FJU:2ZKM-X, respectively. In the first case, as illustrated in Figure 4B with two representatives of the interacting partners of proteins homologous to the QP, the target has two distinct interfaces one of which corresponds to the interface involved in the complex of interest. Unfortunately, T-PIP selected the other one in its prediction. In the second case, Figure 4C, the actual interface of interest does not have a single representative among homologous complexes. As a consequence, T-PIP is not able to make any relevant prediction and suggests the most consensual binding site.

### Ranking Docking Conformations

After generation of possible docking conformations, model scoring allows identifying the most plausible conformation(s). Evaluation of model scoring procedures relies on comparing their ranking of a set of docking conformations with an ‘ideal’ ranking or ‘ground truth’ (GT) generated according to the configuration of the native complex. Since two Capri criteria [78], interface (i-rmsd) and ligand (l-rmsd) rmsds, are appropriate to generate ground truth rankings, here, both were used to produce alternative ground truths for DS93 and DS128 as defined in Table 4 and Figure 2 (See Additional file 5). l-rmsd measures the RMSD between the backbones of the ligand of the predicted complex and the ligand of the actual complex, while i-rmsd restricts its evaluation to interface residues. Comparisons between the GT ranking based on l-rmsd and i-rmsd are shown in Tables 6 and 7. The figures in Tables 6 and 7 are generated by calculating the normalised Pearson's chi-squared statistic (*normalizedχ*^2^) between two different ranking lists. This metric proposed by [70] gives a higher weights to the models that are ranked higher (for details see Methods section); *normalizedχ*^2^ =0 means that the two lists are identical. In the ‘x-rmsd’ column of Tables 6 and 7, ranking generated by one GT criterion (e.g. l-rmsd) is evaluated against the other criterion’s ranking (e.g. i-rmsd). Although *normalizedχ*^2^ values are not 0, they are quite low which means ranking established by the two GT criteria agree quite well with each other. Those values are used as reference scores in further evaluations.

**Table 6:** **Performance of docking model rankings according to ground truth criterion (DS93 dataset) based on average normalized** ***χ***^2^

**Ground truth criterion**	**Ranking method applied to DS93**										
	**x-rmsd**	**Interfaces + PioDock**	**PrISE +PioDock**	**T-PioDock**	**IBIS +PioDock**	**IRAD**	**ZRANK**	**SPIDER**	**SVM**	**TSVM**	**MI**
**i-rmsd**	5.2	11.6	27.5	30.0	30.2	39.5	43.3	49.1	60.7	61.4	67.8
**l-rmsd**	6.0	12.5	27.0	29.7	33.5	39.5	44.2	50.6	63.9	64.5	70.9

In row denoted by i-rmsd and l-rmsd, rankings are compared against ground truth rankings generated by interface rmsd and backbone rmsd, respectively. x-rmsd refers to l-rmsd ranking for the first row and i-rmsd for the second row.

**Table 7:** **Performance of docking model rankings according to ground truth criterion (DS128 dataset) based on average normalized** ***χ***^2^

**Ground truth criterion**	**Ranking method applied to DS128**		
	**x-rmsd**	**Interfaces + PioDock**	**T-PioDock**
**i-rmsd**	5.9	13.6	23.3
**l-rmsd**	6.4	15.0	23.4

In a first set of experiments, T-PioDock was compared to other state-of-the-art methods using DS93. In addition, we evaluate the PioDock module by applying it on the ground truth interfaces of the target complexes (Interfaces + PioDock) instead of their T-PIP predictions. We have used two different metrics to perform this comparison which are (i) *normalizedχ*^2^ and (ii) mean log rank metric (MLR) (for details see Methods section). Table 6 displays the average *normalizedχ*^2^ between the GT and rankings produced by each method. First, although Interfaces + PioDock is not based on interface prediction, but actual interfaces, its *normalizedχ*^2^ is worse than the reference scores (here, it is the double). This can be explained by the fact that since docking interfaces are treated as two set of interface residues without any pairwise knowledge (patches), which is the output of current interface predictors, they could perfectly overlap even if the position of a binding partner was rotated around the centre of the patches. Second, we have investigated usage of other interface predictors (here PrISE and IBIS) along with PioDock (shown as PrISE + PioDock and IBIS + PioDock, respectively) in ranking docking conformations. As demonstrates in the table PioDock based rankings are superior to all other methods whatever the criterion used to generate the GT rankings. Results of PioDock with 3 state-of-the-art template based interface predictors show very similar results (large standard deviations show that differences are not significant). Although those methods generate interfaces with different amino acid compositions, this does not affect PioDock much since it relies implicitly on comparing interface ‘patches’ to see if a complex is compatible or not. These results highlight the robustness of PioDock to small variations in interface predictions. Moreover, relative performances between other methods are in agreement with previously reported results [63,64,67,74].

Analysis of T-PioDock according to its ability to highly rank near-native conformation is performed using the mean log rank metric (MLR) (see Methods section for details). This measures the rank of the first conformation with an RMSD < 10 Å from the actual model. Table 8 displays these results for l-rmsd. Note that since MLR is based on backbone comparison, ranking comparison based on i-rmsd is not possible. Similarly to previous results T-PioDock performs well and all PioDock based rankings of first native conformation improve on other state-of-the-art methods (comparisons using alternative metrics are available in Additional file 6).

**Table 8:** Performance of docking model rankings according to ground truth criterion (DS93 dataset) based on mean log rank metric

**Ground truth criterion**	**Ranking method applied to DS93**									
	**Interfaces + PioDock**	**PrISE +PioDock**	**T-PioDock**	**IBIS +PioDock**	**IRAD**	**SPIDER**	**ZRANK**	**MI**	**TSVM**	**SVM**
**l-rmsd**	1.3	3.1	5.1	5.2	6.5	7.6	8.2	12.3	12.7	13.3

In a second set of experiments, T-PioDock is evaluated on DS128, see Table 7. As expected, better interface predictions for this ‘trivial’ dataset leads to better quality of rankings for T-Piodock compared to DS93.

**Figure 5:**
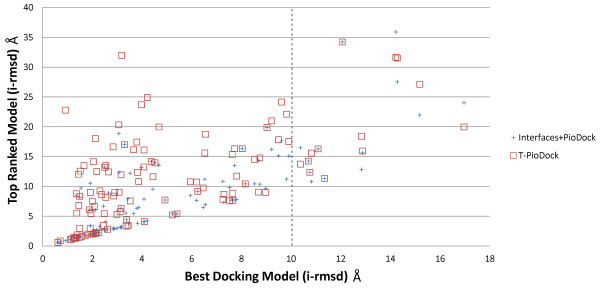
Correlation between the best model produced by docking and the best ranked model according to Interfaces + PioDock and T-PioDock.

In order to have further insight regarding ranking as a mean of identifying near native configurations, Figure 5 displays the i-rmsd of the best produced docking model versus the i-rmsd of the model ranked number one by T-PioDock and Interfaces + PioDock on DS93 and DS128. First, the figure reveals the heterogeneous quality of the best docking model generated for a given target. On this set, the i-rmsd varies between an excellent 0.6 Å to a very poor 17.0 Å with a 4.9 Å average and a large standard deviation of 3.7 Å. Moreover, 13 targets (red squares/blue crosses on the right hand side of the vertical dashed line on Figure 5) did not have a single model below a 10 Å i-rmsd. Second, this figure shows good correlation between the quality of the best docking model and the ability of both T-PioDock and Interfaces + PioDock to detect that model, correlations of 0.65 and 0.81 respectively. A similar pattern is obtained using l-rmsd as GT (See Additional file 7).

**Figure 6:**
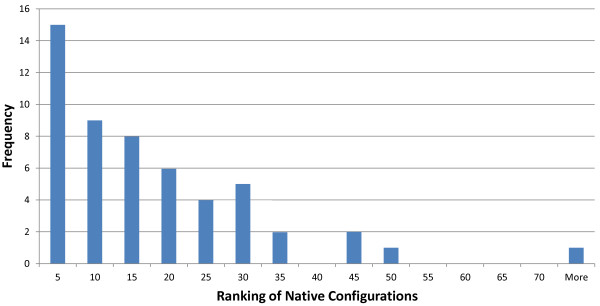
Histogram of the relative T-PioDock rank of the native configuration among all docked models.

Since the quality of the best docking model is very unequal, it is interesting to quantify how it affects model ranking by T-PioDock. In order to study this, best models from the ‘homologous’ target set were clustered using K-means clustering into three groups, i.e. good, average and bad, after normalisation. In Table 9, the average *normalizedχ*^2^ per group shows that T-PioDock produces significant better ranking when a better quality model is available. This suggests that progress in docking would lead to better performance by T-PioDock.Finally, since one of the goals of T-PioDock is to recognise near native models among all predictions, we conducted an experiment where the actual target structure was included in the list of possible models. After ranking, for each target, the relative rank of the native pose among all produced models was extracted. The histogram in Figure 6 shows that the native pose tends to be present in the top of the ranking lists. For example, 16% of the native models are within the 5 first positions.

**Table 9:** **T-PioDock ranking performance (average normalized** ***χ***^2^**based on the quality of the best model**

**Ground truth criterion**	**Quality of the best model**			
	**All**	**Good**	**Average**	**Bad**
**i-rmsd**	30.0	23.7	35.7	39.4
**l-rmsd**	29.7	21.5	37.1	47.6

## Discussion

This study has confirmed that despite sustained activity in the field, the prediction of a complex 3D structure remains a challenge. First, docking software may not be able to produce any near native conformation among the generated set of putative models. Second, identification of the best conformations remains a difficult task. In this work, we have contributed to this topic by offering a pipeline, T-PioDock, for scoring docking models according to the overlap of their components’ predicted interfaces.

Experiments evaluating the proposed scoring process, PioDock, on actual interfaces (Interfaces + PioDock system) showed that the treatment of docking interfaces as patches instead of sets of residue interactions affects the quality of the model selection process: two patches can perfectly overlap even if all binary residue interactions are incorrect. Unfortunately, there is currently no promising alternative since current state of the art in interface prediction is not able to work at such a level of details even if this has started to be explored [61,74]. Although this is an important issue, the study has revealed that the main source of scoring inaccuracy resides with the quality of predicted interfaces, see Table 7. Exhaustive evaluation of interface prediction methods demonstrated that T-PIP is a state-of-the-art method; moreover comprehensive comparison of state-of-the-art methods for ranking docking models supported its integration within the T-PioDock framework. However, as Tables 1 and 2 showed, performance of interface predictions remains unsatisfactory: most metrics returns values within the 40-60% range, with the notable exception of ‘accuracy’ , ~85%, which benefits from the low ratio between interface and non-interface residues. Although there is no doubt that the sustained growth of the PDB [8] will benefit template based methods and T-PIP in particular, Figure 3 also highlighted that T-PIP prediction could not outperform the best available homologue interface. This may be explained by the fact that residues are selected independently without considering pairwise interactions, whereas homologues present interfaces where residues belong to a consistent interaction network. While experiments reported in Table 1 have demonstrated the superiority of template based methods over feature based ones, one would expect than analysis of local features could complement initial template based prediction by bringing local contextual information.

## Conclusion

In this study, we have presented a novel framework, T-PioDock, for prediction of a complex 3D configuration from the structures of its components. It aims to support the identification of near-native conformations by scoring models produced by any docking software. This is achieved by exploiting predictions of complexes’ binding interfaces.

Exhaustive evaluation of interface predictors on standard benchmark datasets has confirmed the superiority of template based approaches and has shown that the T-PIP methodology is a state-of-the-art method. Moreover, comparison between PioDock and other state-of-the-art scoring methods has revealed that the proposed approach outperforms all its competitors.

Despite the fact that detection of native-like models is an active field of research, accurate identification of near-native conformations remains a challenging task. Although availability of 3D complexes will be of benefit to template based methods such as T-PioDock, we have identified specific limitations which need to be addressed. First, docking software are still not able to produce native-like models for every target. Second, current interface predictors do not explicitly refer to binary residue interactions which leaves ambiguity when assessing quality of complex conformations.

## Methods

### T-PIP: Template based Protein Interface Prediction

As described in Figure 1, the T-PIP module, first, evaluates the complexity of a protein target in terms of availability of 3D structures of homologous proteins and, second, applies the most relevant template based interface predictor. In this study, an interface residue is defined according to CAPRI’s definition [44], i.e. an amino acid whose heavy atoms are within 5 Å from those of a residue in a separate chain.

Initially, protein targets are categorised into three categories: ‘trivial’ , ‘homologous’ and ‘unknown’. This is achieved by, first, searching homologues of the query proteins in PDB [8] using Blast [79]. Since the aim is to learn from the interaction pattern of these homologues, only those involved in a complex are further considered. The original target complex under study is purposely removed from the homologue list. In this study, proteins are defined as homologous if their sequence similarity is associated with an Evalue ≤ 10^- 2^. Since predictions are not limited to close homologues as IBIS is [35], interface of more targets can be predicted. If among their homologous complexes both QPs share at least one complex, the target is considered to be ‘trivial’ , since at least a homologue of the complete complex is available. If each QP possesses a set of homologous complexes, but none of them belongs to both sets, the target is classified as ‘homologous’. Finally, if no homologous complex is found for at least one of the QP, the target is judged to be ‘unknown’.

Interfaces of ‘trivial’ targets are simply inferred by aligning the sequence of each QP with the corresponding chain from the ‘best’ common homologous complex and mapping their interface residues on the query chains. In order to select the ‘best’ template among all common complexes, we score them by multiplying the E-values of both components according to their respective QP. The common complex with the lower score is selected as the template from which interfaces are inferred.

**Figure 7:**
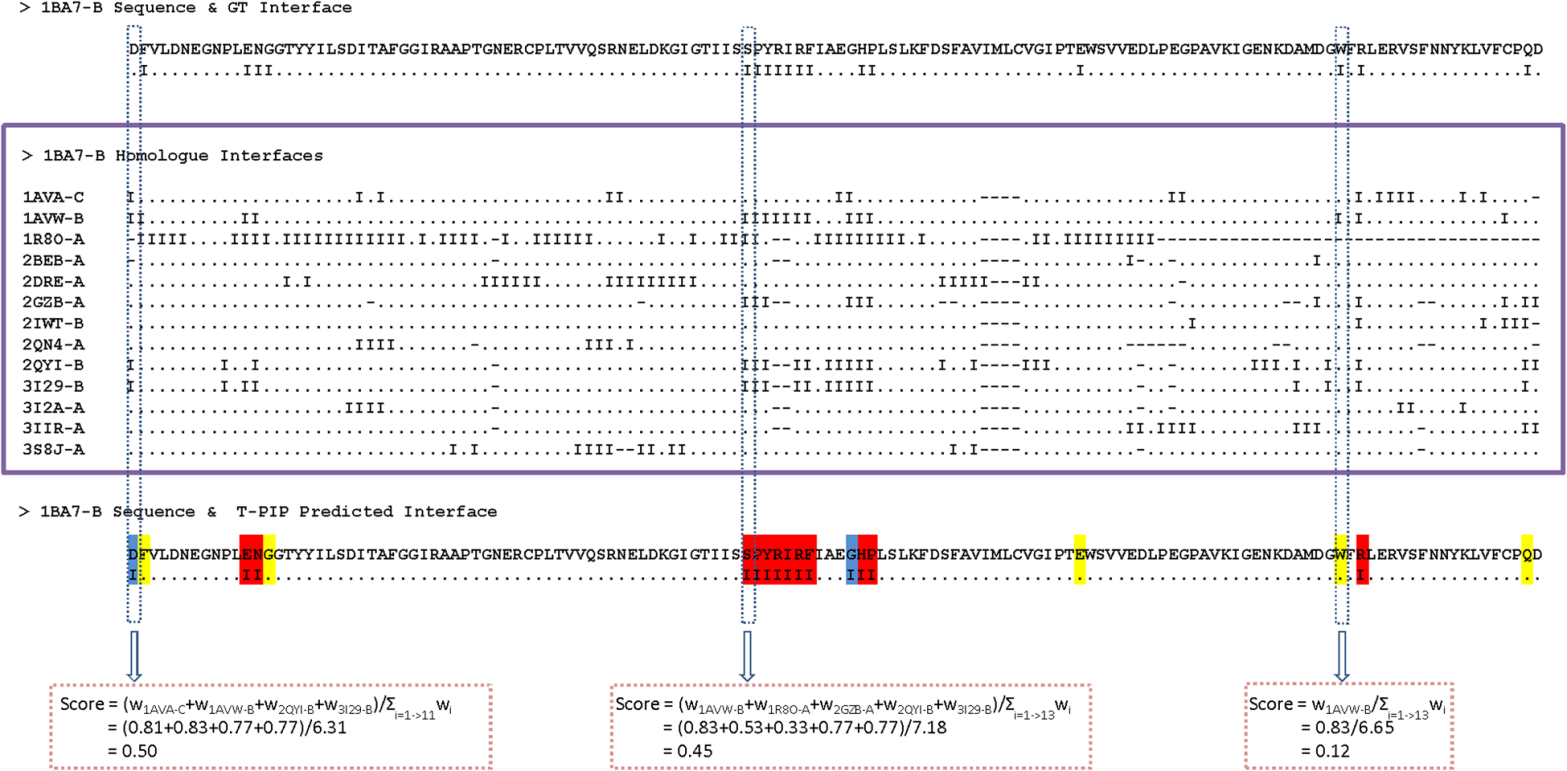
**Example of interface prediction for a ‘homologous’ target.** Red, yellow and blue highlights identify residues which are correctly, missed and wrongly predicted as surface residues. 3D representation of this interface is provided in Figure 4A).

‘Homologous’ targets are processed using the WePIP approach that we proposed recently [32]. For the sake of completeness, it is briefly summarised here. Based on the observation that interface residues are usually structurally conserved between evolutionary related proteins [30], each QP is structurally aligned to its homologous complexes – here we only use the top 30 homologous complexes. Multiple 3D alignment is used to produce a structure-based multiple sequence alignment (S-MSA) in which each residue of the homologue complexes is given a score according to two weights: 1) the query weight is calculated using the homologue E-value against the QP and 2) the ligand weight considers the diversity of the ligand of the homologues. Diversity of ligands is rewarded given that they increase generalisation of interaction patterns. The ligand weight score is designed so that the presence of complex duplicates does not bias predictions towards their configuration. This is done by penalising homologous proteins, whose ligands are similar to each other. Score is calculated as the average sequence identity between the sequence of a ligand and all the others as expressed by the arithmetic mean of the pair wise E-values. Using the scores in the S-MSA, a combined interaction score is calculated for each residues of the QP. Finally, residues with the highest scores are selected as interaction interface (see Figure 7).

Although WePIP was initially designed for predicting interface residues for query proteins whose 3D structure is known, it can also be applied when only the sequence of the query is available. In this case, an initial S-MSA is created using only homologous complexes of the QP. Then, the QP sequence is integrated into that S-MSA using the ClustalW Profile Alignment command [80] to create a complete MSA.

**Figure 8:**
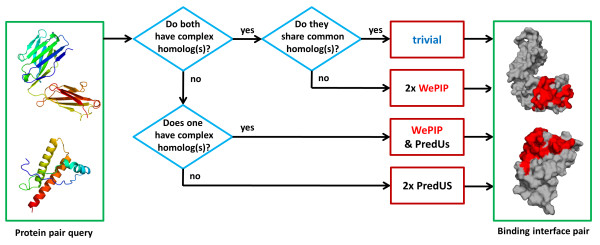
**T-PIP Pipeline.** For a pair of proteins, depending on the existence of homologous complexes, interfaces are categorised as either ‘trivial’, ‘homologous’ or ‘unknown’. The interface of ‘trivial’ is simply predicted using the ‘best’ available complex template. For the ‘homologous’ and ‘unknown’ categories, WePIP and PredUs are used, respectively.

At last, interfaces of ‘unknown’ targets are predicted using a method not relying on homology. As seen in the result section, fewer than 10% of protein targets of the main standard datasets could not benefit from homology based predictions. In this study, we selected a third party template based interface prediction method, PredUs, which has demonstrated good performance [30,32]. Figure 8, summarises the interface prediction pipelines mentioned above.

### PioDock: Protein Interface Overlap for Docking model scoring

PioDock scores docking conformations according to their consistency with interfaces predicted by T-PIP. Given the putative docking conformation of a complex A-B, the model is assigned a score on the basis of the overlap between its interface residues and those predicted for each of its components, i.e. A and B. We define the complex overlap score of A-B complexOverlap_A - B_, as the average between two overlap scores (overlap) calculated for A and B separately:

$${\mathrm{complexOverlap}}_{A-B}=\frac{{\mathrm{overlap}}_{A}+{\mathrm{overlap}}_{B}}{2}$$

where the overlap score for A, overlap_A_, is calculated using the following formula proposed by Kuo et al. [81]:

$${\mathrm{overlap}}_{A}=\frac{\mathrm{interface}\;A_{\mathrm{Docked}}\;\cap \mathrm{interface}\;A_{T-\mathrm{PIP}}}{\sqrt{\left({\mathrm{interfaces}\;A}_{\mathrm{Docked}}\right).\left(\mathrm{interfaces}\;A_{T-\mathrm{PIP}}\right)}}$$

where interface A_Docked_ and interface A_T - PIP_ in the numerator of the formula represent , respectively, the sets of the residue in the interfaces of docked model and the ones predicted by T-PIP. While interface A_Docked_ and interface A_T - PIP_ in the denominator represent the number of residues in the interface of docked model and the ones predicted by T-PIP, respectively.

complexOverlap scores of native complexes should equal to 1, whereas completely incorrect interfaces should be assigned a value of zero. In this study, complexOverlap score was used to rank all conformational models generated by docking software for a given complex. When experiment was conducted to evaluate the PioDock module on its own, actual target interfaces were used instead of their predictions.

Note that when no interface prediction could be performed for one of the two docking partners, the overlap score for that protein is equal to zero and complexOverlap score is calculated using only the overlap score of the other protein.

### Evaluation of docking model scorings

In order to allow any evaluation it is necessary to have some gold standard or ground truth. However, comparison of two docked models is far from being a straightforward task since CAPRI uses three differences measures to assess the docking model quality [78]: l-rmsd measures the RMSD between the backbones of the two complexes ligands, i-rmsd restricts its evaluation to interface residues, whereas *f*_*nat*_ is the fraction of native contacts within the interface. Since *f*_*nat*_ can only discriminate between relatively good configurations – all models failing to predict a single interface residue receive a score of 0, only i-rmsd and l-rmsd are used in this study.

As it was proposed by [70], different model scoring methods can be evaluated by calculating the Pearson's chi-squared statistic between a gold standard ranking of models and rankings generated by those methods. The chi-squared statistic (*x*^2^) determines the goodness of relationship between a set of observed and a set of expected values:

$$\chi^{2}=\sum_{k=1}^{n}\frac{{\left(\mathrm{observe}d_{k}-\mathrm{expecte}d_{k}\right)}^{2}}{\mathrm{expecte}d_{k}}$$

Here, *expected*_*k*_ is the rank of the model κ in the gold standard and *observed*_*k*_ is the rank assigned to model κ by a ranking method. Since the number of docked models may differ between protein pairs, the chi-squared statistic is normalized using the total number of docked models produced for that protein pair, *m*:

$$\mathrm{normalized}\chi^{2}=\frac{\chi^{2}}{m}$$

*normalizedχ*^2^ represents the similarity between two ranking lists by giving higher weights to the models that are ranked higher based on the gold standard: correct ranking is more important for top-ranking models than lower-ranking models. Perfect ranking would return a value of 0.

In order to evaluate if T-PioDock improves the rank of near-native models, we have used mean log rank metric (MLR) introduced in [82]. MLR calculates the mean rank of the first near-native conformation in ranking lists. For each target under study, the rank of the first conformation within 10 Å RMSD deviation of the GT complex (‘hit’) is recorded. Then, the MLR of all targets is calculated as below:

$$\mathrm{MLR}=\exp\left\{\frac{1}{N_{c}}\sum_{i=1}^{N_{c}}\ln\left(\mathrm{Ran}k_{i}\right)\right\}$$

Where *N*_*c*_ is the number of targets and *Rank*_*i*_ is the rank of the ‘hit’ for target *i*. In the best case, if, for all targets, the ‘hit’ is placed in rank 1 then MLR equals to 1.

### Interface prediction evaluation

In order to compare the performance of interface predictors, their True Positive (TP), False Positive (FP), True Negative (TN) and False Negative (FN) rates need to be calculated [83]. Correctness and wrongness of predictions are calculated in respect to the ground truth (GT), which is defined as the X-ray structure of the target protein in its complex form. To summarise these four figures into a single performance measure, a few metrics have been proposed. Below we describe the measures we use in this paper:

To study the quality of predicted interface residues in respect to GT interfaces, recall is used:

$$\mathrm{recall}=\frac{\mathrm{TP}}{\mathrm{TP}+\mathrm{FN}}$$

Recall (also called sensitivity) evaluates the percentage of correctly predicted interfaces. A complement measure to recall is precision which evaluates how many of the predicted interfaces do actually belong to the GT interface:

$$\mathrm{precision}=\frac{\mathrm{TP}}{\mathrm{TP}+\mathrm{FP}}$$

To combine the previous measure, F1 score calculates the harmonic mean of precision and recall:

$$F1=\frac{2\times \mathrm{precision}\times \mathrm{recall}}{\mathrm{precision}+\mathrm{recall}}$$

None of the above mentioned metrics consider the four figures of (TP, TN, FP and FN) at the same time which can bias performance comparison. Therefore, metrics which integrate all the four figures were introduced [84]. Accuracy has been one of the widely used metrics which express the ration of correctly predicted interface and non-interface residues to the total number of cases:

$$\mathrm{accuracy}=\frac{\mathrm{TP}+\mathrm{TN}}{\mathrm{TP}+\mathrm{TN}+\mathrm{FP}+\mathrm{FN}}$$

An alternative has been the Matthews correlation coefficient (MCC):

$$\mathrm{MCC}=\frac{\left(\mathrm{TP}\times \mathrm{TN}\right)-\left(\mathrm{FP}\times \mathrm{FN}\right)}{\sqrt{\left(\mathrm{TP}+\mathrm{FN}\right)\times \left(\mathrm{TP}+\mathrm{FP}\right)\times \left(\mathrm{TN}+\mathrm{FP}\right)\times \left(\mathrm{TN}+\mathrm{FN}\right)}}$$

MCC has shown to be effective especially for predictors which are biased because of the imbalances in their training set.

While receiving operator characteristic (ROC) plots [85] have also been widely used to evaluate classification predictors, they have not been used in this study since very few of our competitors have reported them in their publications.

Since the above mentioned metrics capture different aspects of a predictor’s performance, all of them are required for evaluation.

## Availability and requirements

T-PIP and T-PioDock software are available from http://manorey.net/bioinformatics/wepip/.

## Abbreviations

PDB: Protein Data Bank; MCC: Mathew’s Correlation Coefficient; PQS: Protein Quaternary Structure; MSA: Multiple Sequence Alignment; DBMK: Docking Benchmark; RMSD: Root Mean Square Deviation; QP: Query Protein.

## Competing interests

The authors declare that they have no competing interests.

## Authors’ contributions

JCN proposed the initial concept behind the method. RE implemented the methodology and processed data sets. RE and JCN designed the methodology and performed data analysis. All authors contributed to draft the manuscript. All authors read and approved the final manuscript.

## Supplementary Material

Additional file 1 **Complete list of DS120 targets and their unbound chains.** It provides all the pdb codes of protein targets and their corresponding unbound chains. Click here for file

Additional file 2 **Complete list of DS236 targets and their unbound chains.** It provides all the pdb codes of protein targets and their corresponding unbound chains. Click here for file

Additional file 3 Comparisons between T-PIP, PredUs and PrISE on enzyme-inhibitor, antibody-antigen and others categories of DBMK. Click here for file

Additional file 4 **Complete list of DS93 targets and their unbound chains.** It provides all the pdb codes of protein targets and their corresponding unbound chains. Click here for file

Additional file 5 **Complete list of DS128 targets and their unbound chains.** It provides all the pdb codes of protein targets and their corresponding unbound chains. Click here for file

Additional file 6 **Performance of docking model rankings methods according to ground truth criterion based on Weighted Average Spearman's rank correlation coefficient and Weighted Average Rank of the first solution.** It provides performance comparison (on DS93 dataset) between different ranking methods. Click here for file

Additional file 7 **Correlation between the l-rmsd of best model produced by docking and the best ranked model according to Interfaces + PioDock and T-PioDock.** It displays the l-rmsd of the best produced docking model versus the l-rmsd of the model ranked number one by T-PioDock and Interfaces + PioDock on DS93 and DS128. Click here for file
