# Supplementary material for: Scoring docking conformations using predicted protein interfaces
Source: BMC Bioinformatics. 2014 Jun 6;15:171. doi: 10.1186/1471-2105-15-171 (PMC4057934; doi:10.1186/1471-2105-15-171)
Supplement: Additional file 3 — Comparisons between T-PIP, PredUs and PrISE on enzyme-inhibitor, antibody-antigen and others categories of DBMK. [file 1471-2105-15-171-S3.docx]

Table S1 and Table S2, provide comparisons between T-PIP, PredUs and PrISE on enzyme-inhibitor, antibody-antigen and others categories of DBMK. Based on Table S1, T-PIP performs better in enzyme-inhibitor and others categories. No conclusion can be drawn for antibody-antigen since there is only 1 chain. In Table S2, generally T-PIP shows better performance than PrISE. The exception is in antibody-antigen category in recall, F1 and MCC scores. The reason that T-PIP performance is lower in antibody-antigen category is that T-PIP method involves creating MSA of the homologues. But the formation of anti-body–antigen complexes is different from the standard protein–protein complexes, and therefore it is impossible to create meaningful MSAs (Krawczyk, Baker, Shi, & Deane, 2013).

Table S1: Comparison of T-PIP, PredUs and PrISE on enzyme-inhibitor, antibody-antigen and other categories of DS120.

| **Predictor & Categories** | | **Precision** | **Recall** | **F1** | **Accuracy** | **MCC** |
| --- | --- | --- | --- | --- | --- | --- |
| **T-PIP DS120** | | **52.6** | **56.1** | **52.5** | **85.4** | **45.1** |
|  | **Antibody-Antigen (1 chain)** | 50.0 | 6.7 | 11.8 | 86.5 | 14.5 |
|  | **Enzyme-Inhibitor (47 chains)** | 59.3 | 62.6 | 59.2 | 85.6 | 51.3 |
|  | **Others (72 chains)** | 48.8 | 52.5 | 48.7 | 85.1 | 41.6 |
| **PredUs DS120** | | **47.3** | **58.2** | **48.5** | **69.4** | **24.4** |
|  | **Antibody-Antigen (1 chain)** | 68.4 | 86.7 | 76.5 | 90.8 | 71.6 |
|  | **Enzyme-Inhibitor (47 chains)** | 54.0 | 63.0 | 54.3 | 69.3 | 24.8 |
|  | **Others (72chains)** | 42.5 | 54.5 | 44.2 | 69.2 | 23.5 |
| **PrISE DS120** | | **38.5** | **48.9** | **40.9** | **80.7** | **31.2** |
|  | **Antibody-Antigen (1 chain)** | 39.3 | 68.8 | 50.0 | 80.5 | 41.4 |
|  | **Enzyme-Inhibitor (47 chains)** | 45.2 | 53.2 | 47.2 | 81.7 | 37.2 |
|  | **Others (72 chains)** | 34.1 | 45.9 | 36.6 | 80.1 | 27.1 |

Table S2: Comparison of T-PIP and PrISE on enzyme-inhibitor, antibody-antigen and other categories of DS236.

| **Predictor & Categories** | | **Precision** | **Recall** | **F1** | **Accuracy** | **MCC** |
| --- | --- | --- | --- | --- | --- | --- |
| **T-PIP DS236** | | **53.2** | **55.3** | **52.1** | **85.3** | **44.8** |
|  | **Antibody-Antigen (4 chains)** | 45.0 | 25.4 | 29.3 | 83.6 | 24.4 |
|  | **Enzyme-Inhibitor (90 chains)** | 62.0 | 62.7 | 60.4 | 86.2 | 53.1 |
|  | **Others (140 chains)** | 47.7 | 51.4 | 47.4 | 84.8 | 40.0 |
| **PrISE DS236** | | **41.2** | **47.5** | **41.5** | **81.0** | **32.0** |
|  | **Antibody-Antigen (4 chains)** | 39.7 | 38.4 | 36.9 | 80.7 | 27.2 |
|  | **Enzyme-Inhibitor (92 chains)** | 50.0 | 54.0 | 49.4 | 81.9 | 40.0 |
|  | **Others (140 chains)** | 35.5 | 43.6 | 36.4 | 80.4 | 27.0 |

References:

Krawczyk, K., Baker, T., Shi, J., & Deane, C. M. (2013). Antibody i-Patch prediction of the antibody binding site improves rigid local antibody--antigen docking. *Protein Engineering Design and Selection*, *26*(10), 621–629.
