# Supplementary material for: Scoring docking conformations using predicted protein interfaces
Source: BMC Bioinformatics. 2014 Jun 6;15:171. doi: 10.1186/1471-2105-15-171 (PMC4057934; doi:10.1186/1471-2105-15-171)
Supplement: Additional file 6 — Performance of docking model rankings methods according to ground truth criterion based on Weighted Average Spearman's rank correlation coefficient and Weighted Average Rank of the first solution. It provides performance comparison (on DS93 dataset) between different ranking methods. [file 1471-2105-15-171-S6.docx]

Table S1 displays the Weighted Average Spearman's rank correlation coefficient among different ranking methods on DS93. Correlations close to 1, represent rankings close to the gold standard rankings.

Table S2 displays how well ranking methods rank the best docked model. A Weighted Average Rank of 1 shows that for all targets the ranking method has placed the best model as rank 1.

**Table S1: Performance of docking model rankings according to ground truth criterion (DS93 dataset) based on Weighted Average Spearman's rank correlation coefficient**

| **Ground**  **truth**  **criterion** | **Ranking method applied to DS93** | | | | | | | |
| --- | --- | --- | --- | --- | --- | --- | --- | --- |
|  | **Interfaces +PioDock** | **T-PioDock** | **IRAD** | **ZRANK** | **SPIDER** | **SVM** | **TSVM** | **MI** |
| **i-rmsd** | 0.70 | 0.42 | 0.24 | 0.21 | 0.15 | 0.05 | 0.03 | 0.01 |
| **l-rmsd** | 0.66 | 0.40 | 0.23 | 0.19 | 0.13 | 0.05 | 0.03 | 0.02 |

**Table S2: Performance of docking model rankings according to ground truth criterion (DS93 dataset) based on Weighted Average Rank of the first solution**

| **Ground**  **truth**  **criterion** | **Ranking method applied to DS93** | | | | | | | |
| --- | --- | --- | --- | --- | --- | --- | --- | --- |
|  | **Interfaces +PioDock** | **T-PioDock** | **IRAD** | **ZRANK** | **SPIDER** | **SVM** | **TSVM** | **MI** |
| **i-rmsd** | 5.59 | 14.80 | 19.23 | 21.45 | 24.99 | 35.57 | 35.10 | 40.57 |
| **l-rmsd** | 4.63 | 14.33 | 17.55 | 19.25 | 22.87 | 39.16 | 37.33 | 44.27 |
